# Comparative antimicrobial susceptibility of aerobic and facultative bacteria from community-acquired bacteremia to ertapenem in Taiwan

**DOI:** 10.1186/1471-2334-7-79

**Published:** 2007-07-17

**Authors:** Sai-Cheong Lee, Shie-Shian Huang, Chao-Wei Lee, Chang-Phone Fung, Ning Lee, Wen-Bin Shieh, LK Siu

**Affiliations:** 1 Division of Infectious Diseases, Chang Gung Memorial Hospital, Keelung, Taiwan, Republic of China; 2 Department of Surgery, Chang Gung Memorial Hospital, Keelung, Taiwan, Republic of China; 3 Division of Infectious Diseases, Veterans General Hospital, Taipei, Taiwan, Republic of China; 4 Department of Pathology, Chang Gung Memorial Hospital, Keelung, Taiwan, Republic of China; 5 Department of Chest, Chang Gung Memorial Hospital, Keelung, Taiwan, Republic of China; 6 Division of Clinical Research, National Health Research Institute, Miaoli County,, Taiwan, Republic of China; 7 Chang Gung Institute of Technology, Kwei-Shan Tao-Yuan,, Taiwan, Republic of China; 8 Chang Gung University, Kwei-Shan Tao-Yuan,, Taiwan, Republic of China; 9 National Yang-Ming University, Taipei, Taiwan, Republic of China

## Abstract

**Background:**

Ertapenem is a once-a-day carbapenem and has excellent activity against many gram-positive and gram-negative aerobic, facultative, and anaerobic bacteria. The susceptibility of isolates of community-acquired bacteremia to ertapenem has not been reported yet. The present study assesses the in vitro activity of ertapenem against aerobic and facultative bacterial pathogens isolated from patients with community-acquired bacteremia by determining and comparing the MICs of cefepime, cefoxitin, ceftazidime, ceftriaxone, ertapenem, piperacillin, piperacillin-tazobactam, ciprofloxacin, amikacin and gentamicin. The prevalence of extended broad spectrum β-lactamases (ESBL) producing strains of community-acquired bacteremia and their susceptibility to these antibiotics are investigated.

**Methods:**

Aerobic and facultative bacteria isolated from blood obtained from hospitalized patients with community-acquired bacteremia within 48 hours of admission between August 1, 2004 and September 30, 2004 in Chang Gung Memorial Hospital at Keelung, Taiwan, were identified using standard procedures. Antimicrobial susceptibility was evaluated by Etest according to the standard guidelines provided by the manufacturer and document M100-S16 Performance Standards of the Clinical Laboratory of Standard Institute. Antimicrobial agents including cefepime, cefoxitin, ceftazidime, ceftriaxone, ertapenem, piperacillin, piperacillin-tazobactam, ciprofloxacin, amikacin and gentamicin were used against the bacterial isolates to test their MICs as determined by Etest. For *Staphylococcus aureus *isolates, MICs of oxacillin were also tested by Etest to differentiate oxacillin-sensitive and oxacillin-resistant *S. aureus*.

**Results:**

Ertapenem was highly active in vitro against many aerobic and facultative bacterial pathogens commonly recovered from patients with community-acquired bacteremia (128/159, 80.5 %). Ertapenem had more potent activity than ceftriaxone, piperacillin-tazobactam, or ciprofloxacin against oxacillin-susceptible *S*. *aureus *(17/17, 100%)and was more active than any of these agents against *enterobacteriaceae *(82/82, 100%).

**Conclusion:**

Based on the microbiology pattern of community-acquired bacteremia, initial empiric treatment that requires coverage of a broad spectrum of both gram-negative and gram-positive aerobic bacteria, such as ertapenem, may be justified in moderately severe cases of community-acquired bacteremia in non-immunocompromised hosts.

## Background

Ertapenem is a once-a-day parenteral β-lactam antimicrobial agent [1-6]. Preclinical in vitro studies have shown that this structurally unique carbapenem has excellent activity against many gram-positive and gram-negative aerobic, facultative, and anaerobic bacteria that, in general, are associated with community-acquired infections [1-6]. However, it has minimal activity against *Pseudomonas aeruginosa*, *Acinetobacter *species, and *enterococci *[1-6], and these pathogens usually are associated with nosocomial infections. Recent antimicrobial susceptibility surveillance data indicate that the organisms causing intra-abdominal, skin and soft tissue, and urinary tract infections and community-acquired pneumonia are highly susceptible to ertapenem in vitro [7-17]. However, the susceptibility of isolates of community-acquired bacteremia to ertapenem has not yet been reported. The objective of the present study is to assess the in vitro activity of ertapenem against aerobic and facultative bacterial pathogens isolated from patients with community-acquired bacteremia by determining and comparing the MICs of cefepime, cefoxitin, ceftazidime, ceftriaxone, ertapenem, piperacillin, piperacillin-tazobactam, ciprofloxacin, amikacin and gentamicin. In addition, the prevalence of ESBL producing strains of community-acquired bacteremia and their susceptibility to these antibiotics are investigated.

## Methods

Chang Gung Memorial Hospital at Keelung is an 850-bed(including 55 ICU beds) teaching hospital offering a broad range of services in serving the healthcare needs of about 500,000 residents in northern Taiwan. It has approximately 21,000 admissions per year and mean duration of hospitalization of 8.2 days. Aerobic and facultative bacteria isolated from blood obtained from hospitalized patients with community-acquired bacteremia within 48 hours of admission between August 1, 2004 and September 30, 2004 in Chang Gung Memorial Hospital at Keelung were identified using standard procedures. Community-acquired bacteremia was diagnosed when clinically significant bacterial pathogens were isolated from the blood of hospitalized patients with fever of 38.0≧°C within 48 hours of admission and without admission in past two weeks. Cases with only one blood culture bottle positive for normal skin flora such as coagulase-negative *staphylococci*, *Staphylococcus epidermidis*, *Staphylococcus saprophyticus *and c*orynebacterium *spp. were excluded. All *bacillus*, *micrococcus *and *propionibacterium *isolates which were considered contaminants and anaerobic bacteria were excluded. Clinically significant aerobic bacterial isolates were collected and stored in tryptic soy broth and frozen at -70°C.

Antimicrobial susceptibility was evaluated by Etest according to the standard guidelines provided by the manufacturer and document M100-S16 Performance Standards of the Clinical Laboratory of Standard Institute (CLSI). Antimicrobial agents mentioned above were used against the bacterial isolates to test their MICs as determined by Etest (AB Biodisk, Solna, Sweden). The antimicrobial agents used for each bacteria strain vary from three to eleven (Table 1). For *S. aureus *isolates, MICs of oxacillin were also tested by Etest to differentiate oxacillin-sensitive and oxacillin-resistant *S. aureus*. Detection of *mecA *gene using primers *mec*A1 GTA GAA ATG ACT GAACGT CCG ATA A and *mec*A2 CCA ATT CCA CAT TGT TTC GGT CTA A with polymerase chain reaction method and MICs of amoxicillin/clavulanic acid with Etest for ORSA isolates were also performed using standard procedure. E test was performed on Mueller-Hinton agar (*pneumococci *and *streptococci*: blood agar). The MICs were read at the point where the inhibition ellipse intersected the scale on the strip after incubation at 35°C for 24 hours. Macrocolonies within the ellipse were regarded as significant growth whereas microcolonies could be neglected.

**Table 1:** MIC(μg/ml) breakpoints of susceptibility for each type of bacteria

MIC Breakpoint of Susceptibility (μg/ml)										
Type of bacteria /Antibiotic	FEP	FOX	CAZ	CRO	ERT	PIP	TAZO	CIP	AN	GM
*Staphylococci*	≦8	≦8	≦8	≦8	≦2	≦16	≦8/4	≦1	≦16	≦4
*Streptococci*	≦1	≦8	≦8	≦8	≦1		≦8/4	≦1		
*Enterococci*	≦8				**		≦8/4	≦1		
Enterobacteriaceae	≦8	≦8	≦8	≦8	≦2	≦16	≦16/4	≦1	≦16	≦4
Non-fermenters	≦8	≦8	≦8	≦8	≦2	≦16	≦16/4*	≦1	≦16	≦4

FEP = cefepime, FOX = cefoxitin, CAZ = ceftazidime, CRO = ceftriaxone, ERT = ertapenem, PIP = piperacillin, TAZO = piperacillin-tazobactam, CIP = ciprofloxacin, AN = amikacin GM = gentamicin, MIC = minimal inhibitory concentrations, ≤ indicates less than or equal to.

* For *P. aeruginosa*, MIC susceptibility breakpoint of piperacillin-tazobactam is ≦64/4 μg/ml.

** There are no proposed breakpoints for ertapenem against *enterococci*. In this present study, ≦2 μg/ml was used as breakpoint for ertapenem against *enterococci*.

For quality control, standard control strains were included with each test run. The following organisms with acceptable MICs (μg/ml) limits were included as control strains according to CLSI M7-A7 [18]: *S. aureus *ATCC 29213 (0.12–0.5 for ciprofloxacin, 4–16 for ceftazidime, 1–4 for cefepime, 0.25/4-2/4 for piperacillin/tazobactam), *E. coli *ATCC 25922 (0.004–0.016 for ciprofloxacin, 0.06–0.5 for ceftazidime, 0.016–0.12 for cefepime, 1/4-4/4 for piperacillin/tazobactam), *P. aeruginosa *ATCC 27853 (0.25–1 for ciprofloxacin, 1–4 for ceftazidime, 1–8 for cefepime, 1/4-8/4 for piperacillin/tazobactam), *Streptococcus pneumoniae *ATCC 49619 (0.06–0.25 for cefepime), *Enterococcus faecalis *ATCC 29212 (0.25–2 for ciprofloxacin, 1/4-4/4 for piperacillin/tazobactam).

According to CLSI M7-A7, MIC(μg/ml) breakpoints of susceptibility for each type of bacteria were listed in Table 1.

S. *aureus *isolates with oxacillin MIC of ≦2 μg/ml were regarded as oxacillin-sensitive *S. aureus *(OSSA), whereas isolates with oxacillin MIC of ≧4 μg/ml were regarded as oxacillin-resistant *S. aureus *(ORSA) according to criteria of *S. aureus *susceptibility to oxacillin in CLSI document. M7-A7 published in January 2006. Enterobacteriaceae isolates with ceftazidime MIC ≧2 μg/ml but MIC diminished at least 3 fold after addition of clavulanic acid were regarded as ESBL strains.

## Results and dscussion

There were 70 male and 89 female. Ages ranged from 1 year to 95 years with mean 60.9 years. There were 147 adults (age ≧17 years, range 22–95 years, mean 65.2 years) and 12 pediatric patients (age ≦16 years, range 1–16 years, mean 3.2 years). Of the 159 cases of community-acquired bacteremia, the most likely primary foci of origin according to clinical manifestations are urinary tract infection 59, pneumonia 39, biliary tract infection 17, cellulitis 9, infective endocarditis 3, osteomyelitis 2, peritonitis 1, gastroenteritis 1, and esophagitis 1. The primary foci of origin of the remaining 27 cases of community-acquired bacteremia are unknown. A total of 159 aerobic bacterial pathogens from 159 patients enrolled at Chang Gung Memorial Hospital were collected. Most (95/159, 59.7%) of the isolates were facultative gram-negative bacilli. The most common organism found was *E. coli*, which accounted for 26.4% (42/159) of the total. There were only seven isolates (7/159, 4.4%) with ESBLs (*E. coli *– ESBL, 4; *K*. *pneumoniae *– ESBL, 3).

**Figure 1:**
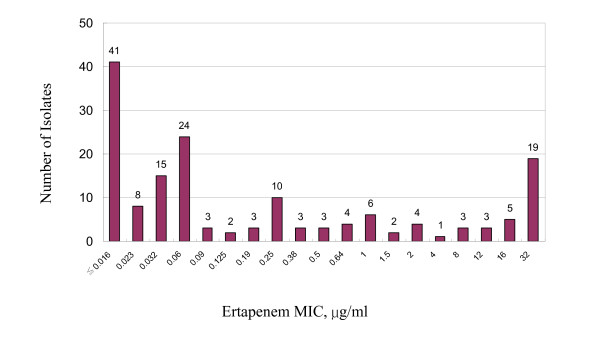
Distribution of ertapenem MIC values for aerobic and facultative bacteria isolated from patients with community-acquired bacteremia.

Figure 1 shows the distribution of ertapenam MIC values for all bacteria isolates tested. Among all the collected isolates, 128 out of 159 isolates (80.5%) were susceptible to ertapenem (MICs ≦2 μg/ml) and 19.5 % was resistant (MICs ≧4 μg/ml). 112 out of 159 isolates (70.4%) were susceptible to ceftriaxone (MICs ≦8 μg/ml). Among the 82 enterobacteriaceae isolates, including the ESBL strains, all (100%) were susceptible to ertapenem while 71 (86.6%) was susceptible to ceftriaxone (Additional file 1). All seven ESBL strains including four *E. coli *-ESBL and three *K. pneumoniae*-ESBL isolates were susceptible to ertapenem, ceftazidime/clavulanic acid and cefotaxime-clavulanic acid (Additional file 1). Seven isolates of coagulase-negative *staphylococci *were repetitively isolated from seven patients and were thus regarded as significant isolates. Three of these seven isolates were susceptible to ertapenem (Additional file 1). Included in the ertapenem-resistant group were *enterococci*, oxacillin-resistant *S. aureus*, oxacillin-resistant coagulase-negative *staphylococci*, *P. aeruginosa *and other non-fermentative gram-negative bacilli (Table 1). Of the two ORSA isolates that were susceptible in vitro to amikacin, one was also susceptible to ciprofloxacin and gentamicin.(Additional file 1). Antimicrobial activities of ertapenem and the comparator agents against all bacteria tested are summarized in Additional file 1. All MICs of control strains obtained with Etest were within the acceptable range listed by CLSI M7A7.

Although most OSSA were susceptible to the majority of agents tested, ertapenem had the most potent activity, based on values for inhibition of 50 % (MIC_50_) and 90% (MIC_90_) of the isolates. All isolates of OSSA were susceptible to ertapenem (17/17, 100 %). Ertapenem was the most active agent against enterobacteriaceae and demonstrated high potency based on MIC_50 _and MIC_90 _values. All isolates of enterobacteriaceae, including most ESBL strains, were susceptible to ertapenem (82/82, 100%) while not as susceptible to ceftriaxone (86.6 %). Among these 159 isolates, there were 38 *E. coli *isolates of which 4 (10.5%) were ESBL strains, and 13 *K. pneumoniae *isolates, of which 3 (23%) were ESBL strains. The prevalence of enterobacteriaceae with ESBLs in community-acquired bacteremia was 7/159, 4.4% in this study. In our hospital, there were 877 significant isolates including fungus, aerobic and anaerobic bacteria causing nosocomial infections in 2006. Among these 877 nosocomial isolates in our hospital in 2006, there were 151 *E. coli *isolates, of which 21 (14%) were ESBL strains, and 69 *K. pneumoniae *isolates, of which 22 (31.8%) were ESBL strains. The overall rate of ESBL strain among nosocomial isolates were 43/877 (4.9%). Although the overall rate of ESBL isolates among community-acquired bacteremia (4.4%) was close to that among nosocomial infections (4.9%), the rate of ESBL isolates among *E. coli *and *K. pneumoniae *were still higher in nosocomial infections. As expected, ertapenem and ceftriaxone had minimal activity against *P. aeruginosa *and *enterococci*. Among the nine ORSA isolates with oxacillin MICs ≧4 μg/ml, two ORSA isolates were *mecA *gene positive with amoxicillin/clavulanic acid MICs 32 μg/ml. The other seven ORSA isolates were *mecA *gene negative with amoxicillin/clavulanic acid MICs range 8–12 μg/ml, mean 11.4 μg/ml, indicating that mechanism of oxacillin resistance in these seven ORSA isolates was not due to *mecA *gene and was most likely due to hyperproduction of β-lactamases [19-22]. Two of the nine ORSA isolates were susceptible to amikacin, including one isolate that was also susceptible in vitro to ciprofloxacin and gentamicin; these two isolates resistant to all β-lactam antibiotics might lack resistance gene to other classes of antibiotics [19-22]. Based on the microbiology of community-acquired bacteremia, empiric treatment requires coverage of a broad spectrum of both gram-negative and gram-positive aerobic bacteria. In this in vitro study, ertapenem was the most active of the agents evaluated against the predominant pathogens – *enterobacteriaceae *and *S*. *aureus*. As anticipated, ertapenem had minimal activity against *P. aeruginosa *and *enterococci*, which was recovered relatively infrequently from community-acquired bacteremic infections. Because of the low frequency of isolation of *P. aeruginosa *and doubtful pathogenicity of *enterococci *in immune competent hosts, initial empiric antimicrobial regimens for bacteremic infections need not include agents specifically directed against these organisms. However, if *P. aeruginosa *is clearly identified as a pathogen in these patients, specific antipseudomonal coverage would be indicated.

## Conclusion

In summary, ertapenem was highly active in vitro against many aerobic and facultative bacterial pathogens commonly recovered from patients with community-acquired bacteremia. Ertapenem had more potent activity than cefepime, cefoxitin, ceftriaxone and piperacillin-tazobactam against methicillin-susceptible *S*. *aureus *and was more active than any of these agents against enterobacteriaceae including strains with ESBL.

Community-acquired bacterial blood stream infections are common in clinical practice. Septicemia secondary to other foci of infections are caused by gram-negative and gram-positive aerobic and facultative bacteria, although *E. coli *and *S. aureus *remain the predominant pathogens. Non-fermentative gram-negative bacteria, which tend to be associated with nosocomial infections, are infrequently involved in community-acquired infections. Based on the microbiology pattern of community-acquired bacteremia, initial empiric treatment that requires coverage of a broad spectrum of both gram-negative and gram-positive aerobic bacteria, such as ertapenem, may be justified in moderately severe cases of community-acquired bacteremia in non-immunocompromised hosts. However, effect of ertapenem on the gut flora is still a concern.

## Authors' contributions

SCL designed the study, carried out the antimicrobial susceptibility studies with Etest and drafted the manuscript. SSH and CWL participated in the antimicrobial susceptibility studies. CPF participated in the design of the study. WBS participated in the statistical analysis. LKS assisted in the detection of *mecA *gene of oxacillin-resistant *S. aureus *with PCR. All authors read and approved the final manuscript.

## Pre-publication history

The pre-publication history for this paper can be accessed here:



## Supplementary Material

Additional file 1 Comparative in vitro activity of ertapenem and comparator agents against aerobic and facultative bacteria isolated from patients with community-acquired bacteremia. Click here for file
