# Supplementary material for: Comparative antimicrobial susceptibility of aerobic and facultative bacteria from community-acquired bacteremia to ertapenem in Taiwan
Source: BMC Infect Dis. 2007 Jul 17;7:79. doi: 10.1186/1471-2334-7-79 (PMC1971056; doi:10.1186/1471-2334-7-79)
Supplement: Additional file 1 — Comparative in vitro activity of ertapenem and comparator agents against aerobic and facultative bacteria isolated from patients with community-acquired bacteremia. [file 1471-2334-7-79-S1.doc]

Comparative in vitro activity of ertapenem and comparator agents against aerobic and facultative bacteria isolated from patients with community-acquired bacteremia.

| **Organism (n)** | **Antibiotic** | **MIC (ug/ml** |  |  | **% Susceptible** |
| --- | --- | --- | --- | --- | --- |
|  |  | **Range** | **MIC50** | **MIC90** |  |
| *Staphylococcus aureus* (OSSA)^a^ (17) | Ertapenem | 0.125-2 | 0.25 | 1.0 | 100 |
|  | Piperacillin | 0.75-64 | 6 | 16 | 88.2 |
|  | Piperacillin/tazobactam | 0.5-12 | 2 | 4 | 94.1 |
|  | Gentamicin | 0.047-64 | 0.5 | 25 | 82.3 |
|  | Amikacin | 1-12 | 3 | 8 | 100 |
|  | Ciprofloxacin | 0.094-0.75 | 0.25 | 0.38 | 100 |
|  | Cefoxitin | 2-64 | 4 | 6 | 88.2 |
|  | Ceftriaxone | 2-48 | 4 | 6 | 88.2 |
|  | Ceftazidime | 8-64 | 16 | 24 | 11.7 |
|  | Cefepime | 1-8 | 3 | 4 | 94.1 |
|  | Oxacillin | :0.38-2.0 | 0.75 | 2.0 | 100 |
| *Staphylocoocus aureus* (ORSA)^b^ (9) | Ertapenem | 12-32 | 32 | 32 | 0 |
|  | Piperacillin | 48-256 | 256 | 256 | 0 |
|  | Piperacillin/tazobactam | 12-256 | 256 | 256 | 0 |
|  | Gentamicin | 4-256 | 256 | 256 | 11.7 |
|  | Amikacin | 1.5-256 | 256 | 256 | 23.5 |
|  | Ciprofloxacin | 0.38-32 | 32 | 32 | 11.7 |
|  | Cefoxitin | 16-256 | 256 | 256 | 0 |
|  | Ceftriaxone | 64-256 | 256 | 256 | 0 |
|  | Ceftazidime | 64-256 | 256 | 256 | 0 |
|  | Cefepime | 12-256 | 256 | 256 | 0 |
|  | Oxacillin | 4.0-256 | 256 | 256 | 0 |
| Coag (-) Staphylococci (7) | Ertapenem | 0.125-32 | 4 |  | 42.8 |
|  | Piperacillin | 1-256 | 6 |  | 57.1 |
|  | Piperacillin/tazobactam | 0.2-256 | 2 |  | 57.1 |
|  | Gentamicin | 0.016-48 | 0.094 |  | 71.4 |
|  | Amikacin | 0.38-16 | 2 |  | 100 |
|  | Ciprofloxacin | 0.125-0.5 | 0.19 |  | 100 |
|  | Cefoxitin | 1-256 | 12 |  | 42.8 |
|  | Ceftriaxone | 0.75-256 | 16 |  | 42.8 |
|  | Ceftazidime | 8-256 | 16 |  | 14.2 |
|  | Cefepime | 1-256 | 4 |  | 71.4 |
| ß-Hemolytic Streptococci ^c^(6) | Ertapenem | 0.032-0.064 | 0.047 |  | 100 |
|  | Ceftriaxone | 0.064-0.125 | 0.125 |  | 100 |
|  | Cefepime | 0.064-0.25 | 0.125 |  | 100 |
| Other Streptococci ^d^(12) | Ertapenem | 0.006-0.38 | 0.032 | 0.38 | 100 |
|  | Ceftriaxone | 0.016-0.38 | 0.094 | 0.125 | 100 |
|  | Cefepime | 0.064-0.75 | 0.094 | 0.5 | 100 |
| *Enterococcus* spp^e,f^ (11) | Ertapenem | 0.032-32 | 8 | 12 | 27.2 |
|  | Piperacillin | 1-16 | 4 | 16 | 100 |
|  | Piperacillin/tazobactam | 1-16 | 4 | 16 | 54.5 |
|  | Gentamicin | 0.38-256 | 16 | 256 | 45.4 |
|  | Amikacin | 3-256 | 256 | 256 | 27.2 |
|  | Ciprofloxacin | 0.19-32 | 1 | 6 | 54.5 |
| *Escherichia coli* (38) | Ertapenem | 0.004-1.5 | 0.016 | 0.094 | 100 |
|  | Piperacillin | 0.75-256 | 256 | 256 | 26 |
|  | Piperacillin/tazobactam | 0.016-256 | 1.5 | 3 | 92 |
|  | Gentamicin | 0.1-256 | 1 | 192 | 71 |
|  | Amikacin | 1-8 | 2 | 4 | 100 |
|  | Ciprofloxacin | 0.006-32 | 0.125 | 12 | 84 |
|  | Cefoxitin | 1.5-256 | 4 | 48 | 82 |
|  | Ceftriaxone | 00.016-256 | 0.032 | 4 | 92 |
|  | Ceftazidime | 0.016-48 | 0.125 | 0.38 | 95 |
|  | Cefepime | 0.016-4 | 0.125 | 0.5 | 100 |
| *Escherichia coli*－ESBL (4) | Ertapenem | 0.016-1 | ≦0.1 |  | 100 |
|  | Piperacillin | 16-256 | ≦256 |  | 25 |
|  | Piperacillin/tazobactam | 0.032-3 | ≦1.5 |  | 100 |
|  | Gentamicin | 1-256 | ≦2 |  | 75 |
|  | Amikacin | 2-256 | ≦8 |  | 75 |
|  | Ciprofloxacin | 0.032-1 | ≦0.75 |  | 100 |
|  | Cefoxitin | 3-256 | ≦256 |  | 50 |
|  | Ceftriaxone | 12-256 | ≦64 |  | 0 |
|  | Cefepime | 0.19-256 | ≦4 |  | 75 |
|  | Ceftazidime | 2-32 | ≦32 |  | 50 |
|  | Ceftazidime-clavulanic acid | 0.064-4 | ≦4 |  | 100 |
|  | Cefotaxime | 0.26-16 | 16 |  | 25 |
|  | Cefotaxime/clavulanic acid | 0.016-1 | ≦1 |  | 100 |
| *Klebsiella pneumonine* (13) | Ertapenem | 0.012-1.9 | 0.016 | 0.064 | 100 |
|  | Piperacillin | 2-256 | 6 | 256 | 53.8 |
|  | Piperacillin/tazobactam | 1-2 | 2 | 2 | 100 |
|  | Gentamicin | 0.19-256 | 1 | 1.5 | 85 |
|  | Amikacin | 1-256 | 2 | 16 | 92 |
|  | Ciprofloxacin | 0.016-12 | 0.125 | 2 | 92 |
|  | Cefoxitin | 2-8 | 3 | 6 | 100 |
|  | Ceftriaxone | 0.023-0.064 | 0.032 | 0.064 | 100 |
|  | Ceftazidime | 0.094-0.38 | 0.125 | 0.38 | 100 |
|  | Cefepime | 0.032-0.19 | 0.047 | 0.19 | 100 |
| *Klebsiella pneumoniae* — ESBL (3) | Ertapenem | 0.016-1.9 | ≦0.5 |  | 100 |
|  | Piperacillin | 256 | 256 |  | 0 |
|  | Piperacillin/tazobactam | 256 | 256 |  | 0 |
|  | Gentamicin | 32-256 | 256 |  | 0 |
|  | Amikacin | 2-256 | 256 |  | 33.3 |
|  | Ciprofloxacin | 0.125-2 | ≦1.5 |  | 33.3 |
|  | Cefoxitin | 32-256 | 32 |  | 0 |
|  | Ceftriaxone | 256 | 256 |  | 0 |
|  | Ceftazidime | 32 | 32 |  | 0 |
|  | Cefepime | 24-256 | 24 |  | 0 |
|  | Ceftazidime-clavulanic acid | 0.19-4 | ≦4 |  | 100 |
|  | Cefotaxime | 16 | 16 |  | 0 |
|  | Cefotaxime/clavulanic acid | 0.094-0.38 | 0.25 |  | 100 |
| *Aeromonas hydrophilia* (4) | Ertapenem | 0.008-0.23 | ≦0.032 |  | 100 |
|  | Piperacillin | 3-256 | 256 |  | 25 |
|  | Piperacillin/tazobactam | 0.75-2 | 0.75 |  | 100 |
|  | Gentamicin | 1-256 | 256 |  | 25 |
|  | Amikacin | 2-6 | ≦6 |  | 100 |
|  | Ciprofloxacin | 0.016-0.032 | ≦＜0.032 |  | 100 |
|  | Cefoxitin | 4 | 4 |  | 100 |
|  | Ceftriaxone | 0.016-0.094 | 0.016 |  | 100 |
|  | Ceftazidime | 0.125-0.38 | 0.125 |  | 100 |
|  | Cefepime | 0.064-0.125 | 0.125 |  | 100 |
| *Proteus mirabilis* (9) | Ertapenem | 0.019-0.094 | 0.032 | 0.094 | 100 |
|  | Piperacillin | 0.25-256 | 256 | 256 | 33 |
|  | Piperacillin/tazobactam | 0.25-1 | 0.5 | 1 | 100 |
|  | Gentamicin | 1-256 | 256 | 256 | 33 |
|  | Amikacin | 2-8 | 4 | 8 | 100 |
|  | Ciprofloxacin | 0.016-3 | 0.064 | 3 | 67 |
|  | Cefoxitin | 3-6 | 4 | 6 | 100 |
|  | Ceftriaxone | 0.016-1.5 | 0.016 | 1.5 | 100 |
|  | Ceftazidime | 0.016-0.25 | 0.094 | 0.25 | 100 |
|  | Cefepime | 0.094-1.5 | 0.125 | 1.5 | 100 |
| *Morganella morganii* (3) | Ertapenem | 0.016-0.064 | 0.047 |  | 100 |
|  | Piperacillin | 0.38-256 | 3 |  | 66.6 |
|  | Piperacillin/tazobactam | 0.25-1 | 0.38 |  | 100 |
|  | Gentamicin | 1-96 | 2 |  | 67 |
|  | Amikacin | 1.5-4 | 3 |  | 100 |
|  | Ciprofloxacin | 0.012-1.5 | 0.032 |  | 67 |
|  | Cefoxitin | 8-256 | 16 |  | 33 |
|  | Ceftriaxone | 0.016-0.5 | 0.25 |  | 100 |
|  | Ceftazidime | 0.094-8 | 0.5 |  | 100 |
|  | Cefepime | 0.023-0.19 | 0.032 |  | 100 |
| *Salmonella* spp. (4) | Ertapenem | 0.016-0.032 | ≦0.032 |  | 100 |
|  | Piperacillin | 1.5-256 | 256 |  | 25 |
|  | Piperacillin/tazobactam | 1.5-4 | ≦4 |  | 100 |
|  | Gentamicin | 0.38-256 | ≦16 |  | 50 |
|  | Amikacin | 0.38-1.5 | 1.5 |  | 100 |
|  | Ciprofloxacin | 0.016-12 | ≦2.0 |  | 50 |
|  | Cefoxitin | 2-8 | ≦3 |  | 100 |
|  | Ceftriaxone | 0.016-0.064 | 0.047 |  | 100 |
|  | Ceftazidime | 0.125-0.38 | 0.25 |  | 100 |
|  | Cefepime | 0.064-0.19 | 0.064 |  | 100 |
| Miscellaneous Enterobacteriaceae^g^ (6) | Ertapenem | 0.012-0.064 | 0.047 |  | 100 |
|  | Piperacillin | 0.125-256 | 4 |  | 66.6 |
|  | Piperacillin/tazobactam | 0.125-2 | 1 |  | 100 |
|  | Gentamicin | 0.5-256 | ≦1.5 |  | 83.3 |
|  | Amikacin | 1-3 | ≦2 |  | 100 |
|  | Ciprofloxacin | 0.008--32 | ≦0.125 |  | 83.3 |
|  | Cefoxitin | 2-256 | ≦6 |  | 66.6 |
|  | Ceftriaxone | 0.032-256 | 1 |  | 83.3 |
|  | Ceftazidime | 0.047-0.75 | ＜0.125 |  | 100 |
|  | Cefepime | 0.023-4 | 0.19 |  | 100 |
| *Branhamella catarrhalis* (1) | Ertapenem | 0.032 |  |  | 100 |
|  | Ceftriaxone | 0.064 |  |  | 100 |
|  | Cefepime | 0.094 |  |  | 100 |
| *Pseudomonas aeruginosa* (5) | Ertapenem | 32 | 32 |  | 0 |
|  | Piperacillin | 3-256 | 64 |  | 80 |
|  | Piperacillin/tazobactam | 3-256 | 96 |  | 60 |
|  | Gentamicin | 2-256 | 16 |  | 40 |
|  | Amikacin | 4-48 | 4 |  | 80 |
|  | Ciprofloxacin | 0.125-32 | 3 |  | 40 |
|  | Cefoxitin | 2-256 | 256 |  | 20 |
|  | Ceftriaxone | 12-256 | 256 |  | 0 |
|  | Ceftazidime | 1-256 | 128 |  | 40 |
|  | Cefepime | 2-256 | 128 |  | 40 |
| Miscellaneous Nonfermenters^h^ (5) | Ertapenem | 2-32 | 16 |  | 20 |
|  | Piperacillin | 16-256 | 256 |  | 20 |
|  | Piperacillin/tazobactam | 0.016-256 | 48 |  | 40 |
|  | Gentamicin | 1-256 | 256 |  | 40 |
|  | Amikacin | 1-256 | 4 |  | 80 |
|  | Ciprofloxacin | 0.125-32 | 6 |  | 60 |
|  | Cefoxitin | 2-256 | 256 |  | 20 |
|  | Ceftriaxone | 3-256 | 48 |  | 20 |
|  | Ceftazidime | 0.75-256 | 8 |  | 80 |
|  | Cefepime | 0.5-256 | 6 |  | 80 |
| *Pseuodomonas* spp. (1) | Ertapenem | 32 |  |  | 0 |
|  | Piperacillin | 48 |  |  | 0 |
|  | Piperacillin/tazobactam | 32 |  |  | 0 |
|  | Gentamicin | 0.75 |  |  | 100 |
|  | Amikacin | 4 |  |  | 100 |
|  | Ciprofloxacin | 0.25 |  |  | 100 |
|  | Cefoxitin | 32 |  |  | 0 |
|  | Ceftriaxone | 16 |  |  | 0 |
|  | Ceftazidime | 16 |  |  | 0 |
|  | Cefepime | 256 |  |  | 0 |
| *Leuconostoc* spp. (1) | Ertapenem | 0.047 |  |  | 100 |
|  | Ceftriaxone | 0.19 |  |  | 100 |
|  | Cefepime | 0.125 |  |  | 100 |

Key:^a^OSSA, oxacillin-susceptible *S. aureus,*

^b^ORSA, oxacillin-resistant *S. aureus*,

ESBL, extended spectrumβ-lactamase.

^c^β-hemolytic streptococci includes 5β-hemolytic Group B streptococci and 1β-hemolytic Group non-ABD

streptococci.

^d^Other Streptococci includes 2 *Streptococcus anginosus*, 2 *Streptococcus constellatus*, 2 *Streptococcus equisimilis*,

2 *Streptococcus mitis*, 1 *Streptococcus bovis*, 1 *Streptococcus pneumoniae*, 1 *Streptococcus vestibularis*, 1

viridans streptococcus.

^e^ Ceftriaxone was considered ineffective against enterococci.

^f^*Enterococcus* spp. includes 9 *Enterococcus faecalis*, 2 *Enterococcus faecium*.

^g^Miscellaneous Enterobacteriaceae includes 1 *Citrobacter freundii*, 1 *Enterobacter cloacae*, 1 *Klebsiella oxytoca*,

2 *Proteus vulgaris*, 1 *Providencia akalifaciens*.

^h^Miscellaneous nonfermenters includes 2 *Acinetobacter baumannii*, 2 *Acinetobacter lwoffii*, 1 *Burkholderia cepacia*.
